# Supplementary material for: Spontaneous single-nucleotide substitutions and microsatellite mutations have distinct distributions of fitness effects: Distributions of fitness effects of spontaneous mutations
Source: bioRxiv. 2023 Jul 4:2023.07.04.547687. Preprint. [Version 1] doi: 10.1101/2023.07.04.547687 (PMC10349969; doi:10.1101/2023.07.04.547687)
Supplement: Supplement 1 [file media-1.pdf]

Supporting Information

S1 File. S1_file_S1_file_PIE_setup_file.csv. *PIE* setup file used for image analysis.

S2 File. YFR054C-Scw11p-GFP-Cyc1T-HERP1.1_delitto_perfetto.str. Sequence of *YFR054C* locus after insertion of full GFP marker cassette and hygromycin selection.

S3 File. S3_file_YFR054C-Scw11p-GFP-Cyc1T-post-dp.str. Sequence of *YFR054C* locus with GFP marker cassette after FuDR counterselection and resistance cassette removal.

S1 Table. Estimated mutational effect of each 2000-generation mutation accumulation strain. The mutational effect *s* and petite proportion for the single haploid 2000-generation MA progeny phenotyped in this study (MA.H lines), as well as two haploid lines derived from the 2000-generation mutation accumulation ancestor (MA.T.0.a1-a2). 95% confidence interval presented in parentheses.

S2 Table. Properties of DMEs identified by various models using mutational effect summary data. Preliminary models fitted to summary data of individual strain *s* value fits. Models that attempt to fit unidentified mutations either as additional mutations drawn from the same reflected gamma distribution as SNMs, or as an independent reflected gamma distribution to individual unidentified mutations, improve fit only marginally. The '*two-gamma*' model produces uninformative parameter estimates, with poor computational likelihood estimation across the likelihood profile (due to confounded parameters). The resulting non-monotonic likelihoods lead to uninterpretable confidence interval bound estimation for many parameters (question marks). The 'Gaussian' model, which accounts for unidentified mutations as a Gaussian distribution representing the effects of these mutations across strains, performs better than a model that only accounts for SNMs. Parameter values for each model shown with 95% Confidence Intervals; ΔAIC is calculated relative to the "SNMs only" model.

S3 Table. Estimated mutational effect of each *msh3*Δ mutation accumulation strain. The mutational effect *s* and petite proportion for *msh3*Δ MA strains phenotyped in this study, as well as the $MSH3^{+}$ strain from which the *msh3*Δ ancestor was derived (a clone of *MAT0.a1*), and a GFP-marked *msh3*Δ strain used as an in-well reference. 95% confidence interval presented in parentheses. % genome sequenced at >10x and not part of a repetitive sequence comes from data in [38].

S4 Table. Variables used in modeling. Variables used in modeling the DME or individual strain *s* values

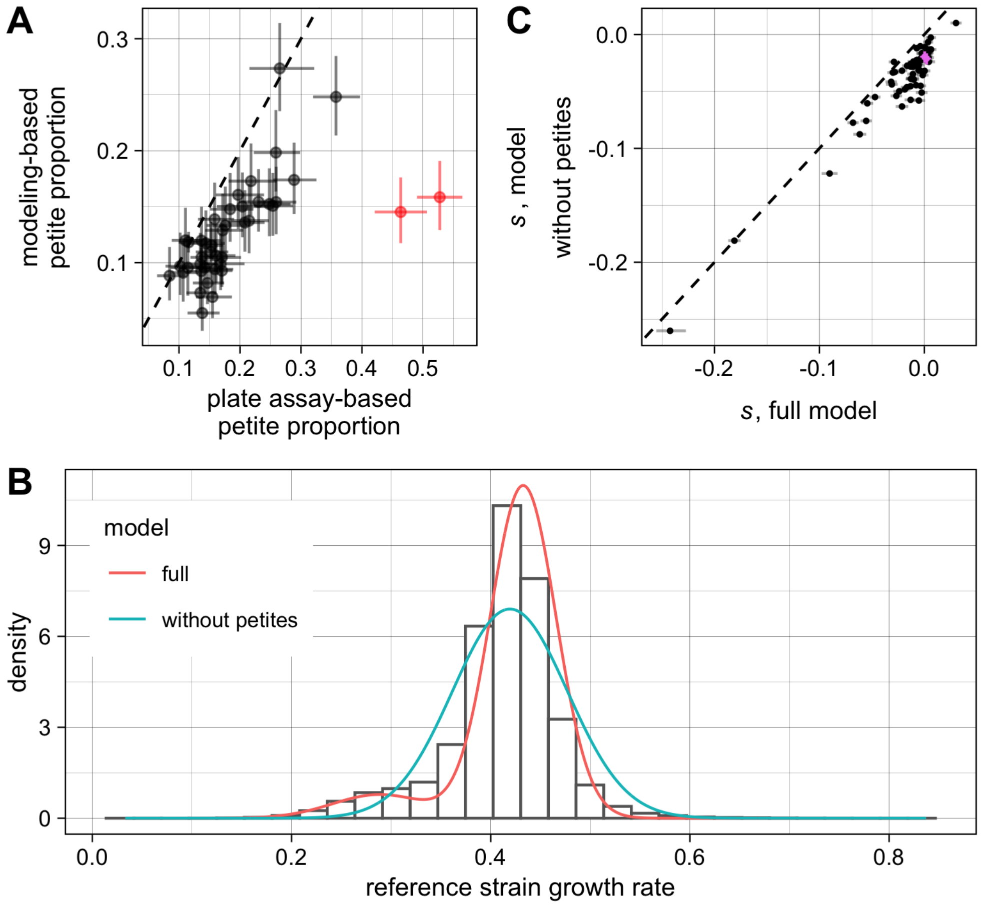

S1 Fig

(A) Petite proportion estimated for strains from [38] using plate-based colony color assay versus by modeling observed colony growth rates in the microscope assay as a mixture of Gaussians. Error lines represent 95% confidence intervals, dashed line shows a 1:1 correspondence. Each point represents estimates for a single strain on a single experimental day (with error bars based on replicates across microscope plate wells). Two data points in red are for a strain in which colony color and colony size were decoupled.

(B) A histogram of growth rates of the ancestral reference strain in all wells in which it was co-cultured with 2000-generation *MAH* strains. Red line shows the distribution estimated by the best-fit model of the distribution of growth rates for this strain in the full model (including a distribution of petites); blue line shows distribution estimated by the model in which no separate petite distribution is included. Note that although distributions are shown overlaid on raw reference strain growth rate measurements, the models that produced the distribution parameters were based on differences between reference strain and MA strain growth rates (see Methods).

(C) Mutational effects for strains from Fig 1 estimated either by the full model of MA strain *s* effects described in the text, or by a model that does not include a petite population in any of the strains. Error lines represent 95% confidence intervals, dashed line shows a 1:1 correspondence. Two ancestral control strains included in the experiments (purple points) have mutational effects whose confidence intervals overlap with 0 when petites are accounted for, but not when they are ignored.

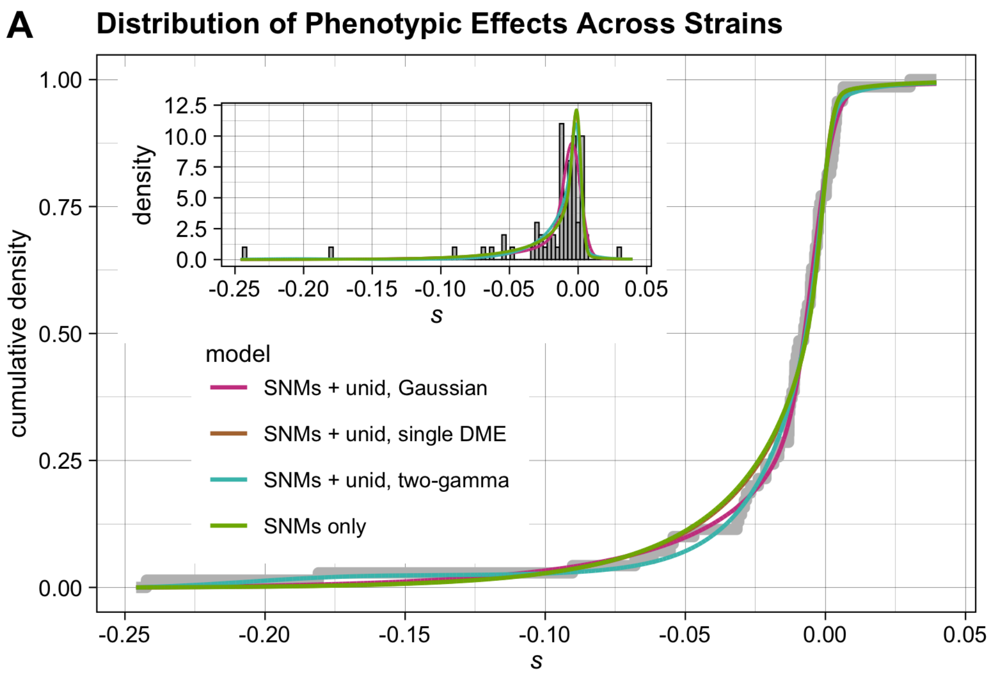

S2 Fig

(A) The cumulative density function of the fit of each DME model to all individual MA strain mutational effects *s*. Inset: histogram of mutational effects with probability density functions of the models overlaid. To account for the effect of experimental noise on the estimates of *s*, the model density function is shown convolved with a Gaussian noise kernel with a variance that is the mean of the error variances of each strain's mutational effect estimate. *SNMs + unid, Gaussian* model as in **Fig 2**
